# Silicone Arthroplasty as an Alternative to Arthrodesis in the Metatarsophalangeal Degenerative Disease of Hallux Valgus—A 5-Year Observational Study

**DOI:** 10.3390/jcm13133677

**Published:** 2024-06-24

**Authors:** Jędrzej Lesman, Joanna Wojna, Patrycja Szkutnik, Bartłomiej Tomasik, Marcin Domżalski, Przemysław Łaganowski

**Affiliations:** 1 Department of Orthopedics and Traumatology, University Clinical Hospital WAM, Żeromskiego 113, 90-549 Łódź, Poland; wojenka.j@gmail.com (J.W.); patrycja.szkutnik@wp.pl (P.S.); marcin.domzalski@umed.lodz.pl (M.D.); przemlag@poczta.onet.pl (P.Ł.); 2 Department of Oncology and Radiotherapy, Faculty of Medicine, Medical University of Gdańsk, 80-210 Gdańsk, Poland; bartlomiej.tomasik@gumed.edu.pl

**Keywords:** foot, mtp1, arthrodesis, arthroplasty, osteoarthritis

## Abstract

**Background**: Arthroplasty is gaining more and more popularity in the treatment of osteoarthritis (OA) of the metatarsophalangeal I joint (MTP1). The aim of our study was to evaluate the early and long-term objective clinical and radiographic results, as well as the subjective results, of MTP1 arthroplasty in comparison to MTP1 arthrodesis among patients with OA and a valgus deformity of their MTP 1 joint. **Methods**: Patients with OA MTP1 were examined before surgery and in the 5-year period after surgery. The inclusion criteria for the study were OA of the MTP1 joint and a non-axial position of the toe in valgus between 20 and 40 degrees. Prostheses were created for the patients with higher demands for mobility of their MTP 1 joint and arthrodesis was carried out for those with lower expectations. The treatment outcomes were assessed by clinical examination, radiography, the AOFAS scale, the SEFAS scale, and using patient-related outcome measures (PROM). **Results**: A total of 39 people, 37 women and 2 men, aged 55 to 67 years old (average, 61 years old) participated in the study. During the follow-up period, there were no complications in the form of infection or a loosening of the implant after both arthroplasty and arthrodesis. The follow-up examination 60 months after the surgery showed an improvement in scores (>20 points) on both the AOFAS and SEFAS scales. All patients, after surgery, reported reduced pain. **Conclusions**: The use of a silicone prosthesis in the surgical treatment of degenerative changes in the MTPI joint, with appropriate indications and excluding cases with large hallux valgus, gives better results than arthrodesis.

## 1. Introduction

Osteoarthritis (OA) in the forefoot is the most often located in the first metatarsophalangeal joint (MTP I.) and less frequently in the remaining metatarsophalangeal joints, i.e., in the joints of the same row. OA MTP I occurs in about 2% of people over 50 and is manifested as joint pains and the formation of periarticular bone growths leading to reduced mobility in the MTP I joint [1]. Due to the progressive deformation and stiffness of the joint, this disease is most often referred to as hallux rigidus (big toe). In the initial stage of the development of OA MTP I, conservative treatment is usually undertaken (shoe inserts, painkillers, and anti-inflammatory drugs), but as the deformity develops and symptoms worsen, patients require surgery. The most commonly used surgical methods were traditionally cheilectomy (the removal of bone spikes on the dorsal side of MTP I), osteotomy of the metatarsal and/or proximal phalanx, arthrodesis (joint stiffening), or arthroplasty with the excision of the articular surfaces [2]. The coexistence of hallux valgus in the OA of MTP 1 is a challenging factor for the future surgery. Stiffening of the MTP I joint is still the standard treatment for OA; however, it leads to a shortening of the first ray of the foot, which in turn may reduce the patient’s gait efficiency and cause pain [3].

When performing arthrodesis in the metatarsophalangeal joint, the force transmission system changes during gait, both within the first radius and the lateral column of the foot, which leads to the development of degenerative changes in the tarsus and hindfoot [3]. In recent years, arthroplasties of the MTP I joint are performed more and more often, especially in younger and active people. The aim of the operation is to relieve pain, restore the mobility and stability of the joint, and consequently restore its biomechanics and physiological gait [3]. For the proper placement of the prosthesis, it is necessary to resect the distal part of the metatarsal bone and the proximal part of the basal phalanx (of the first toe). 

Hallux valgus can be a difficult challenge for the surgeon due to problems with the proper congruence of the joint. The occurrence of two diseases at the same time means that the patient expects us to correct both of them at the same time.

The use of silicone endoprostheses is a solution that can be administered to all MTP joints, and also in patients with poorer bone quality, e.g., in the course of osteoporosis and rheumatoid diseases [4]. The short- and medium-term results of MTP joint arthroplasty with the use of silicone prostheses are usually satisfactory; in over 80% of patients pain relief or significant relief is noted and the amplitude of movement improves to about 30 degrees [5].

## 2. Materials and Methods

### 2.1. Study Design

This study was created as a prospective, two-cohort trial. Upon receiving consent from our host university’s Bioethical Committee to perform this project, the patients were qualified for the study. The study was planned as a long-term observational study based on patient-related outcomes. This study was accepted by the Medical University of Łódź’s Ethical Committee, with number RNN/158/15/KE, on 26 September 2015. All steps of the study were conducted in accordance with the Declaration of Helsinki and the STrengthening the Reporting of OBservational studies in Epidemiology (STROBE). A checklist for cross-sectional studies was downloaded from www.equator-network.org (accessed on 2 February 2024). 

### 2.2. Participants

The study participants were patients qualified for the surgery in the Clinic of Orthopedics in Veterans’ Memorial Hospital in Łódź. The subjects voluntarily agreed to participate in the study. The main inclusion criteria were symptomatic MTP 1 arthritis with a grade of 3 or 4 on the Coughlin scale and a hallux valgus angle between 20 and 40 degrees in radiographs. Moreover, a clinical presentation of pain and willingness to undergo the surgery were essential for patients to participate in the study. The exclusion criteria were previous fractures or surgery of the MTP 1 joint, as well as patients with osteoporosis, rheumatoid diseases, and severe hallux valgus (a hallux valgus angle over 40 degrees). The eligibility criteria were met by 39 consecutive patients who had an arthritic MTP 1 joint. The data were obtained from 2015 to 2023. There were 37 females and 2 males included, with a mean age of 63.9 ± 10.2 years old (range, 48–72 years old). The average follow-up was 60 months ±1 month. One patient was not reported in the follow-up because of their death. No other patients were lost during follow-up. All participants were well informed and provided written consent.

### 2.3. Surgery 

Patients were assigned to each type of surgery depending on their wish for the movement of their MTP I joint after the surgery. The patients who were willing to preserve their propulsion were offered the prosthesis of their MTP I joint. The other choice was arthrodesis. The patients were presented with all of advantages and disadvantages of arthrodesis and arthroplasty. After this final qualification, the patients received foot X-rays (AP and view) in order to obtain repeatability in the angle analysis. The patients’ surgeries were performed by one surgeon (PŁ), with the assistance of the other co-authors of this article. Both surgeries were performed through a lateral approach. The arthroplasty was performed using Wright™ (Memphis, TN, USA) implants according to the manufacturers manual. The arthrodesis was carried out using Arthrex™ (Munich, Germany) plate with screws. The methods and implants used for the surgery were consequently the same for each group.

### 2.4. Data Collection

The X-ray was the main tool used for radiological qualification and in the follow-up criteria. The hallux valgus angle (HVA); intermetatarsal angle (IMA); lateral subluxation of the sesamoid, according to the scale of the AOFAS Research Committee; and the grade of arthritis, in terms of the Manz classification, were assessed. The measurements were performed by two observers (JL and PS). Intraclass correlation coefficients (ICCs) for the echogenicity measurements were calculated to assess intra- and inter-rater reliability. The ICCs for intra-rater agreement ranged from 85% to 97% for Rater 1 and from 87% to 95% for Rater 2, indicating excellent reliability. For inter-rater agreement, the ICCs ranged from 80% to 97%, with these values indicating moderate to excellent reliability.

The following demographic and anthropometric variables were recorded: age, sex, weight, height, and body mass index (BMI). Patients were obliged to fill out the AOFAS and SEFAS scales before the surgery. The patients then underwent a standardized operation for their prosthesis (*n* = 21) or first MTPJ arthrodesis (*n* = 18).

All patients were seen postoperatively in the outpatient clinic at 1, 3, 6, 12, 36, and 60 months after the surgery by the main surgeon. Patient-related outcomes were also obtained during these follow-up visits by the main surgeon’s assistants (JL, PS, JW) directly from the patients.

### 2.5. Bias

To prevent potential sources of bias, stringent inclusion and exclusion criteria were applied to both cases and controls. To prevent a failure in the assessment of scales, the same team of orthopedic surgeons was used to control the patients’ outcomes. There is also a possibility of hypothetical bias being present within the surgery. However, this bias was mitigated by ensuring that all of the surgeries were conducted consistently by a single researcher (PŁ) using uniform procedures.

### 2.6. Study Size

Our a priori sample size estimate was based on the results from a pilot study performed on the first 10 procedures in each group [6]. This approach was introduced due to the fact that the effect size could not be determined—there was a lack of previous similar studies. G*Power-3.1 software was utilized (Heinrich-Heine-Universität Düsseldorf, Düsseldorf, Germany).

### 2.7. Statistical Analysis

The statistical analysis was performed by one of the authors (BT). Qualitative variables are presented as percentages. The chi-square test with appropriate corrections was used for the analysis. Continuous variables are presented with their median and interquartile range. The normality of the distribution was verified with the Shapiro–Wilk test. Intergroup differences for continuous variables were analyzed using the Mann–Whitney U-test for independent pairs. In order to assess the correlation between the variables, the Pearson rank correlation was used. Linear regression modules and generalized linear models were used in a multivariate analysis aimed at determining the independent risk factors for complications. A repeat-measure ANOVA was used for the analysis of more than 2 groups. All statistical calculations were performed using Statistica 13.3 software (StatSoft, Cracow, Poland).

## 3. Results

First, a comparison between the two groups (arthrodesis vs. arthroplasty) was performed. No statistically significant differences were observed between two groups. The results are presented in Table 1.

The patients that underwent arthrodesis were older than the arthroplasty patients (66 years old vs. 61 years old), but this difference did not reach statistical difference.

No complications were observed in the postoperative period.

Taking into the consideration the degree of arthritis in the MTP joint, no statistical difference was observed. The same number of patients with grade II arthritis qualified for arthrodesis and arthroplasty as those with grade III.

An important result that was observed was the degree of sesamoid lateral subluxation. Arthrodesis was performed in patients with a higher grade of subluxation than those in the second group. Statistical significance was observed between all of the groups (*p* < 0.049). The results are presented in Table 2.

The difference between the AOFAS and SEFAS scales did not reach statistical significance before the surgery. The mean result on the SEFAS scale for both groups was 19 (SD = 4), the mean result on the AOFAS scale for arthroplasty was 40 (SD = 9), while it was 37 for arthrodesis.

**Figure 1:**
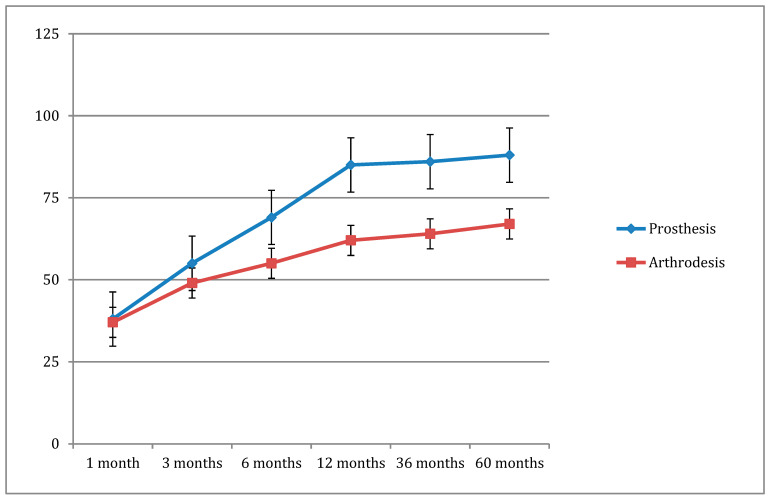
The declining trends in SEFAS scores.

The scales were analyzed in the post-operative period. The main result which was observed was a decline in the SEFAS scale. The later the visit, the lower the result observed (Figure 1). This finding was observed equally and with equal dynamics in both groups. The result was not affected by the HVA, IMA, or sesamoid subluxation. 

**Figure 2:**
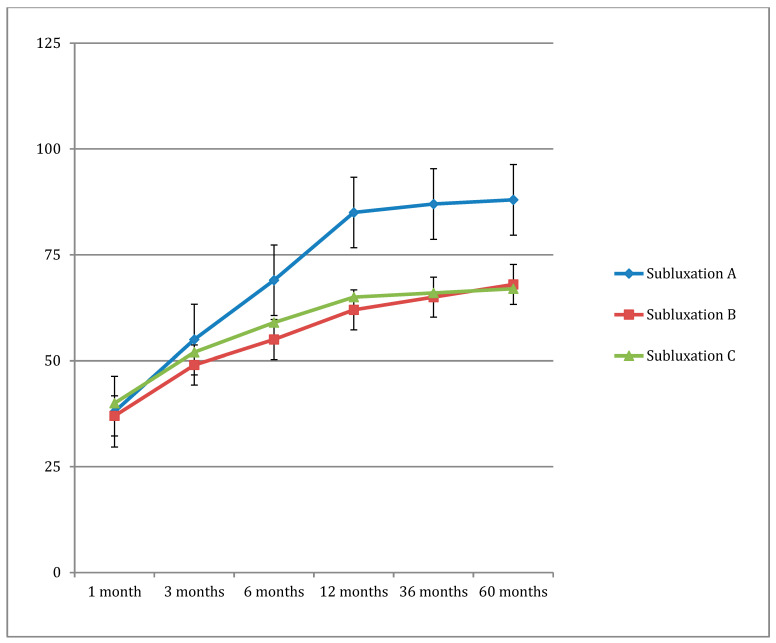
Correlation between sesamoid subluxation and the increase in AOFAS scores.

On the AOFAS scale, a gradually increasing score was observed. In this case, however, a statistically significant correlation was observed with the HVA and subluxation in both groups. In the group with A grade subluxation, the increase in the AOFAS scale score was higher than in groups with the other types of subluxation (Figure 2).

In both scales, the main result was achieved one year after the surgery. The next observations presented a slight moderation in the results, with a trend of improvement on both scales. However, the comparison between 12, 36, and 60 months after the surgery did not reach statistical significance.

The hallux valgus angle was correlated with a higher increase in AOFAS scale scores (*p* < 0.05). This trend is not associated with the IMA. No statistical associations were observed with the BMI.

## 4. Discussion

A large clinical trial showed that arthroplasty of the first MTPJ had success rates comparable to first MTPJ arthrodesis, regardless of the patients’ gender, age, or BMI. Although the degrees of the HVA and IVA were higher in the group receiving arthrodesis, the moderate cases treated either with arthroplasty or arthrodesis provided similar results in later scales. This finding suggests that alloplastics of the MTP I joint could be useful for patients who expect propulsion after the surgery and generally have higher demands.

First metatarsophalangeal joint arthroplasty can preserve foot gait and maintain biomechanics more closely to their natural state. Although earlier MTP implants had unsatisfactory outcomes, advancements in our understanding foot biomechanics have led to the creation of improved implants with better clinical outcomes and longevity. A meta-analysis of 3049 MTP 1 joint replacements, with an average follow-up period of 61 months, reported a 94.5% satisfaction rate post-surgery [7].

The literature typically suggests that joint-sparing procedures are best for mild to moderate osteoarthritis, while fusion is recommended for late-stage moderate to severe osteoarthritis. However, our findings contradict this view, as we observed no significant difference in outcomes between the groups, regardless of the degree of hallux valgus [8]. 

The main difficulty for the researchers was comparing the outcomes for osteoarthritic hallux valgus with those in previous studies, especially when hallux valgus is present and the hypermobility plays an important role in the development of osteoarthritis. Former studies present mostly arthroplasty or arthrodesis for typical cases of hallux rigidus. The osteoarthritic part of the disease is taken into consideration rather than the bony structure (hallux valgus) [9,10].

In the current study, both groups showed significant improvements in their pain, functional, and clinical scores. This aligns with the recent literature on MTP 1 joint replacements. Although some studies have compared the outcomes of 1MTP arthrodesis and arthroplasty, most focus on older-generation prostheses, which have been linked to less favorable results [7,11,12].

Synthetic-implant-first MTPJ arthroplasty can effectively treat patients with mild hallux valgus. However, patients with a hallux valgus greater than 40 degrees were excluded from the clinical trial, as concurrent valgus correction procedures were not allowed. Therefore, we cannot provide outcomes for first MTPJ replacements in cases with hallux valgus exceeding 40 degrees [13].

According to a recent meta-analysis, one of the main reported benefits of interposition arthroplasty is an improved range of motion. The review noted that the range of motion improved in all studies, with results comparable to those for a total joint replacement or resurfacing arthroplasty. Additionally, our study’s results are consistent with this and support arthroplasty as a viable treatment option.

In comparison to other long-term studies, the number of cases presented here are higher and the follow-up is strict and simple [14]. The AOFAS and SEFAS scales are an effortless form of control for the patients and present clear results in the postoperative period.

The important part of this research were the correlations between the scales and radiological findings. It has been proven that larger hallux valgus angles can interfere with the results of patients’ satisfaction after a surgery for hallux rigidus [15]. However, our study presented no interaction between the BMI and final results. According to this, the contraindications for prosthesis are narrowing and this could be a solution for patients regardless of obesity.

The main strengths of this paper are that it is a comparative study in which all patients were operated on by the same surgeon using a standardized technique. The surgeon had extensive experience with these procedures, which likely contributed to the low complication rate. Additionally, the follow-up for patients in the arthroplasty group was relatively high, and no additional complications were identified despite thorough clinical and radiographic evaluations.

The presented study also has some limitations. Firstly, the sample size is relatively small, and the primary assessment criteria rely on clinical examinations and scales. In response, the authors acknowledge that innovative and long-term observation should be simplified to yield clear results. Additionally, patients were not randomly assigned to each group but rather selected for prosthesis or fusion based on their first ray’s alignment and their patient characteristics. However, the authors believe that this patient selection process is crucial to the success of the techniques.

## 5. Conclusions

In conclusion, our five-year follow-up data suggest that synthetic arthroplasty is a suitable treatment option for patients with hallux valgus and comparable to arthrodesis. Our findings indicate that it was a viable option for hallux rigidus cases accompanied by mild hallux valgus angles (≤30 degrees), particularly in patients with greater post-surgical demands. There were no specific contraindications noted.

## Figures and Tables

**Table 1:** The demographic comparison of arthrodesis vs. arthroplasty.

	Median (25–75%) Arthroplasty	Median (25–75%) Arthrodesis	*p*
BMI	25.4 (23.8–27.3)	25.9 (24.2–28.2)	0.66
HVA	14.0 (12.0–20.0)	20.0 (15.0–32.0)	0.23
IMA	9.0 (8.0–11.0)	13.0 (9.0–18.0)	0.42

**Table 2:** The grade of subluxation (A—medium, B—high, C—highest).

	Grade A	Grade B	Grade C
Arthroplasty	15	3	3
Arthrodesis	7	5	6

## Data Availability

The data are contained within the article.
